# A Multi-sectoral Institutional Action to Boost Integrated Antimicrobial Stewardship to Curtail Antimicrobial Resistance: A World Antimicrobial Resistance Awareness Week (WAAW) Initiative in North India

**DOI:** 10.7759/cureus.102482

**Published:** 2026-01-28

**Authors:** Prasan K Panda, Satyasree B, Maneesh Sharma, Girraj Saini, Ashutosh Sharma, Rakhi Mishra, Gaurav Chikara, Mahendra Gehlot, Bhupinder Solanki, Rupinder Deol, Amber Prasad, Sukriti Yadav, Pushpa Rani, Amit Kumar, Jino Jacob, Jeetendra K Sharma, Nikhil B, Sowjanya Perumalla

**Affiliations:** 1 Medicine, All India Institute of Medical Sciences Rishikesh, Rishikesh, IND; 2 Pediatric Surgery, All India Institute of Medical Sciences Rishikesh, Rishikesh, IND; 3 Nursing, All India Institute of Medical Sciences Rishikesh, Rishikesh, IND; 4 Pharmacology, All India Institute of Medical Sciences Rishikesh, Rishikesh, IND; 5 Social and Preventive Medicine, All India Institute of Medical Sciences Rishikesh, Rishikesh, IND; 6 Microbiology, All India Institute of Medical Sciences Rishikesh, Rishikesh, IND; 7 Nursing Administration, All India Institute of Medical Sciences Rishikesh, Rishikesh, IND; 8 Pharmacy, All India Institute of Medical Sciences Rishikesh, Rishikesh, IND

**Keywords:** amr, antimicrobial resistance, antimicrobials, antimicrobial stewardship, awareness, india, integrated antimicrobial stewardship (ias), waaw, world antimicrobial resistance awareness week

## Abstract

During World Antimicrobial Resistance Awareness Week (WAAW) 2024, All India Institute of Medical Sciences (AIIMS) Rishikesh implemented a structured, multi-sectoral Institutional Action Plan (IAP) to transition from conventional awareness activities to an integrated antimicrobial stewardship approach. The initiative emphasized coordinated institutional action through education, advocacy, and practice-based interventions involving healthcare workers, hospital administration, and the community. The seven-day program included institutional capacity building, assessment and incentivization, and public and community outreach activities. Evaluation was conducted using predefined indicators assessing healthcare worker knowledge enhancement through pre- and post-intervention assessments, compliance with bedside antimicrobial stewardship practices via interactive audits, adoption of standardized prescribing tools, and community outreach through educational roleplays and talks. Targeted training was delivered to over 300 healthcare workers, including focused stewardship training for pharmacists. The program achieved institution-wide engagement involving more than 3,000 participants. Key antimicrobial stewardship policy documents were released during the inauguration, reflecting administrative commitment. Bedside stewardship activities audited and trained over 1,000 healthcare workers across more than 40 clinical areas, leading to the identification of a high-performing clinical unit as the institutional stewardship champion. Core stewardship practices were reinforced through an institutional pledge taken by over 2,000 healthcare workers, and individual stewardship champions were identified across professional categories. Structured workshops strengthened stewardship competencies among selected participants, while public outreach activities reached over 1,500 community members. Media engagement further amplified program visibility and messaging. The IAP implemented during WAAW 2024 demonstrated a feasible, scalable, and integrated institutional model for antimicrobial stewardship. By combining policy reinforcement, structured training, performance recognition, and community engagement, the initiative fostered measurable behavior change across institutional and public domains. This technical framework may be adapted by other tertiary-care institutions to strengthen antimicrobial stewardship implementation and sustain long-term efforts to contain Antimicrobial Resistance (AMR).

## Introduction

Antimicrobials, the foundational therapeutic pillars of modern medicine, comprising antibiotics, antifungals, antivirals, and anti-parasitic drugs, have profoundly altered the landscape of human health and surgical capability [1]. Yet, the efficacy of these vital agents is being systematically eroded by the accelerated, inevitable evolutionary consequence of their widespread application: Antimicrobial Resistance (AMR) [2]. The pervasive use of antimicrobials in human, animal, and environmental contexts, often for prophylactic, therapeutic, and growth promotion purposes, drives the selection and proliferation of multidrug-resistant organisms (MDROs), or "superbugs" [3]. The resulting global health crisis is no longer a future projection but a present reality, characterized by escalating treatment failures, prolonged hospitalization, and sharply increasing mortality rates [4]. The severity of the AMR threat is quantified by recent, robust epidemiological data. The 2022 Global Burden of Disease (GBD) Study on AMR estimated that in 2019 alone, bacterial AMR was directly responsible for 1.27 million deaths and associated with 4.95 million deaths worldwide [5]. The escalating trajectory of resistance projects catastrophic consequences. While the 2016 O'Neill review is often cited, recent projections reaffirm the trajectory, estimating that the global economic toll could reach 100 trillion by 2050, largely driven by the anticipated rise in mortality [6]. This existential threat positions AMR alongside climate change as one of the most pressing global challenges of the century [7].

In response to this urgency, global governance bodies have formalized critical, time-bound objectives: The 79th United Nations General Assembly (UNGA) high-level meeting on AMR established a clear, political commitment to achieving a 10% reduction in human deaths related to bacterial AMR by 2030 [8]. The World Health Organization (WHO)’s Global Action Plan (GAP) on AMR continues to guide the 'One Health' approach, requiring all member states to develop and implement current National Action Plans (NAPs) that integrate human, animal, and environmental sectors [9]. In the United States, the Centers for Disease Control (CDC)’s AMR Solutions Initiative is focused on strengthening domestic infrastructure for surveillance, laboratory capacity, and effective infection prevention and control (IPC) across all healthcare and community settings [10]. In the Indian context, the National and State Action Plans (NAPs/SAPs) provide the overarching framework. However, a critical implementation gap persists in translating these macro-level mandates into concrete, measurable, and standardized Institutional Action Plans (IAPs) within individual healthcare facilities, the primary epicenters of antimicrobial prescribing and resistance selection [11].

The annual World AMR Awareness Week (WAAW) serves as an essential, global mobilization platform to stimulate and recognize intervention efforts. The WAAW 2024 theme, "Educate. Advocate. Act now," provided the impetus for the development of an innovative, high-impact IAP at AIIMS Rishikesh designed to transition from fragmented stewardship to a cohesive, Integrated Antimicrobial Stewardship (IAS) framework. Our primary objective was to institutionalize a multi-sectoral IAS framework through a seven-day IAP that bridges the gap between global AMR policy (UNGA 2030 targets) and bedside clinical practice at a tertiary-care setting. This holistic model transcends mere prescription review, encompassing rigorous IPC measures, optimization of diagnostic stewardship, and continuous, data-driven feedback on antimicrobial utilization [12]. The secondary objectives are capacity building, policy & digital integration, behavioral change and public health advocacy. This initiative demonstrates that the IAP model is a critical sentinel operational unit, a microcosm of the national strategy that creates an immediate, boosting effect on IAS practices among the patient population, healthcare workers (HCWs), and the surrounding community. It reaffirms our institutional commitment to leading the regional response in curtailing the AMR crisis through data-driven, scalable, and sustainable interventions thereby fulfilling our institutional responsibility in the global fight to curtail AMR.

## Technical report

Material and methods

The WAAW 2024 program was executed from November 18 to 24, 2024, by the Antimicrobial Stewardship Program (AMSP) committee, utilizing a multi-disciplinary team of faculty, residents, nurses, pharmacists, hospital attendants (HA), housekeeping (HK) staff, guards, students, patients and their caretaker population. This was a prospective, quasi-experimental, multi-modal educational intervention and implementation study conducted over a seven-day period. The week-long activities, designed to reach both institutional stakeholders and the general public (Table 1), were structured around three core pillars.

**Table 1:** The WAAW 2024 program schedule with details of the total participants The operational roadmap and participant distribution for the World Antimicrobial Resistance Awareness Week (WAAW) 2024 Institutional Action Plan

S.No	Date and day	Activities	Venue	Total participants (n)
1	18/11/2024 Monday	Inauguration with an oath ceremony and the release of various guidelines	Lecture theatre -3 (LT-3)	141
		Workshop on Integrated Antimicrobial Stewardship (IAS) Practices to create Training Of Trainers (TOT)		98
2	19/11/2024 Tuesday	Role plays by Nursing Officers (NOs) on hand hygiene (first play), isolation (second play), and biomedical waste (BMW) segregation (third play) to curtail Antimicrobial resistance (AMR) among the public	3 out-patient department (OPD) counters	174
		Ice-breaking sessions with healthcare workers (HCWs) in 15 areas and identifying the IAS champion ward/Intensive Care Unit (ICU) using a checklist	Ward/ICUs	256
		Role plays in school; awareness talk in school	PM Shri SSSNS Atal Utkrisht Government Inter College, Thano, Dehradun	250
		Quiz competition	LT-3	30
3	20/11/2024 Wednesday	3 role plays by hospital attendants (HA), housekeeping staff (HK), Medical Records Clerk (MRC) (one each) on hand hygiene (first play), environment cleaning (second play), and adult vaccination (third play) to curtail AMR among the public	3 OPD counters	159
		Ice-breaking sessions with HCWs in 15 areas and Identifying IAS champion ward/ICU by checklist	Wards/ICUs	182
		Role plays in school; awareness talk in school	Government Inter College, Raiwala, Dehradun	250
		Quiz competition	LT-3	40
4	21/11/2024 Thursday	3 role plays by BSc Nursing, Paramedical, and MBBS students	3 OPD counters	176
		Ice-breaking sessions with HCWs in 12 areas and Identifying IAS Champion ward/ICU by checklist	Wards/ICUs	126
		Role plays in school; awareness talk in school; poster competition in school	Government Inter College, IDPL, Veerbhadra, Rishikesh	300
5	22/11/2024 Friday	Quiz/poster competition on WAAW theme for nurses, residents, students	LT-3	40
		Identifying final IAS champions in each category	Dept. of Micro & Pharma	80
		Foundation workshop on IAS Practices for residents and nurses	Mini-auditorium	92
		Ice-breaking sessions with HCWs in 12 areas and identifying IAS champion ward/ICU by checklist	Wards/ICUs	114
6	23/11/2024 Saturday	Pharmacy Stewardship Workshop	LT-B	60
		Closing ceremony		220
7	24/11/2024 Sunday	Community awareness activity with local pharmacists	Local pharmacy shops	50-60
		Roleplay (on AMR impact with death) with a talk (How can AMR be effectively contained?) at a public gathering	Triveni Ghat	160
	Everyday (18-24^th^)	Awareness on social media	YouTube, WhatsApp	Many

Institutional capacity building

Inauguration and Policy Release

The week commenced with an inauguration and oath ceremony (Table 2) on day one of WAAW where the institutional commitment to right diagnosis, mandatory IPC, judicious antimicrobial use was formalized.

**Table 2:** AIIMS Rishikesh Institutional Action Plan (IAP) oath AIIMS Rishikesh's institutional mobilization and professional commitment via the antimicrobial stewardship (AMS) pledge. AIIMS, All India Institute of Medical Sciences; ASMP, Antimicrobial Stewardship Program.

Oath Ceremony	
As a healthcare professional, I recognize that antimicrobials are vital in saving lives. I also acknowledge the alarming rise of antimicrobial resistance, threatening global health.	
I (We/Hospital) Pledge to:	
	Accept antimicrobial resistance is rising, and I am here to decrease it.
	Promote integrated antimicrobial stewardship practices by preventing and controlling infections, doing right investigation and treatment for right patient at right time.
	Foster source control and always follow standard precautions for all patients.
	Audit my hand hygiene and environment cleaning practices.
	Advocate and use adult vaccination.
	Adhere to facility-specific treatment recommendations for infectious diseases based on national guidelines and local antibiograms.
	Use standard operating procedures for specimen collection, storage, and transportation.
	Prescribe or use antimicrobials judiciously, considering the diagnosis, severity, and local resistance patterns.
	Restrict to backfoot the reserved antimicrobials.
	Optimize antimicrobial use through regular documentation and review.
	Educate myself, my family members, patients and their caregivers on infectious diseases, their chain of transmission, and their cure with antimicrobials.
	I acknowledge one health, caring for others, I care for myself.
By taking this pledge, I commit to being a responsible healthcare professional and protecting human beings from antimicrobial resistance. Regards: AMSP Committee, AIIMS Rishikesh.	

Four critical policy documents prepared by the AMSP committee were officially released which were: (1) disease-specific updated infectious diseases (ID) guidelines and antimicrobial audit forms; (2) antibiogram and specimen request form (SRF) for diagnostic standardization; (3) antimicrobial stewardship interventions document (including duration of therapy, redundancy lists, intravenous (IV)-oral switch protocols, outpatient parenteral antimicrobial therapy (OPAT), and pharmacokinetics - pharmacodynamics (PK-PD) guidance); and (4) launch of the dedicated AMSP section on the All India Institute of Medical Sciences (AIIMS) Rishikesh website for HCWs and public.

Foundation Workshops

Dedicated three-hour workshops on IAS practices were conducted for newly joined residents, consultants, and nurses.

Pharmacy Stewardship Workshop

This session focused on equipping pharmacists with tools to manage antimicrobial inventory, including the use of the WHO AWaRe list utility, consumption analysis, and the implementation of a critical sub-alert system for reserve group antimicrobials.

Training assessment and incentivization of HCWs

IAS Checklist-Based Audit

'Ice-breaking' sessions were deployed daily on day two to five of WAAW by expert teams (consisting of a consultant, assistant nursing superintendent, and resident or senior nurse) in all wards and ICUs (Appendix). The team was given a random area based on lottery system, thereby minimizing inter-rater variability. The selected team went to the area and did an assessment-cum-training on the IAS practices. Multi-disciplinary teams utilized a comprehensive IAS checklist to assess adherence to local administration (availability of policy documents, dashboard displays, IPC (hand hygiene, environment cleaning, biomedical wastage (BMW) management), diagnostic stewardship practices (sample collection, documentation), AMSP practices (appropriate documentation, IV-oral switch adherence). Discrepancies were documented, and on-the-spot positive feedback and training were provided.

IAS Championship Awards

To incentivize adherence, a competition was held to identify two types of IAS champions: 1) IAS champion per ward/area (assessed via the checklist score given by the expert team and later verified by the administrative expert team), and 2) An individual IAS champion among the consultants, nurses, resident doctors, interns, pharmacists, HAs, HK staff, security guards, and students (medical, nursing para-medical), who were assessed via Multiple Choice Questions (MCQ) and structured interviews.

Public and community outreach

Roleplays and Skits

Different roleplays were performed throughout the week by students and HCWs. These performances delivered simplified, high-impact messages on hand hygiene, respiratory hygiene, BMW segregation, patient isolation, adult vaccination, and the necessity of prescriptions for antimicrobials in three busy out-patient department (OPD) areas (days two to four of WAAW), three local government schools (days four to six) and a final performance (day seven) at the public gathering site of Triveni Ghat (spiritual meeting place on the belt of river Ganga where thousands get together for prayer).

School and Community Awareness

Awareness talks were delivered to all available students across three higher secondary schools, emphasizing basic infection prevention strategies and antimicrobial uses. Poster competitions were also organized.

Media Engagement

Active social media campaigns, institutional displays, and coverage in local newspapers were utilized throughout the week to disseminate AMR awareness and AMR prevention to a broader public audience.

The Kirkpatrick Model of Evaluation was applied to categorize our metrics, moving from participation (Level 1) to behavioral change (Level 3). 

Results

**Figure 1:**
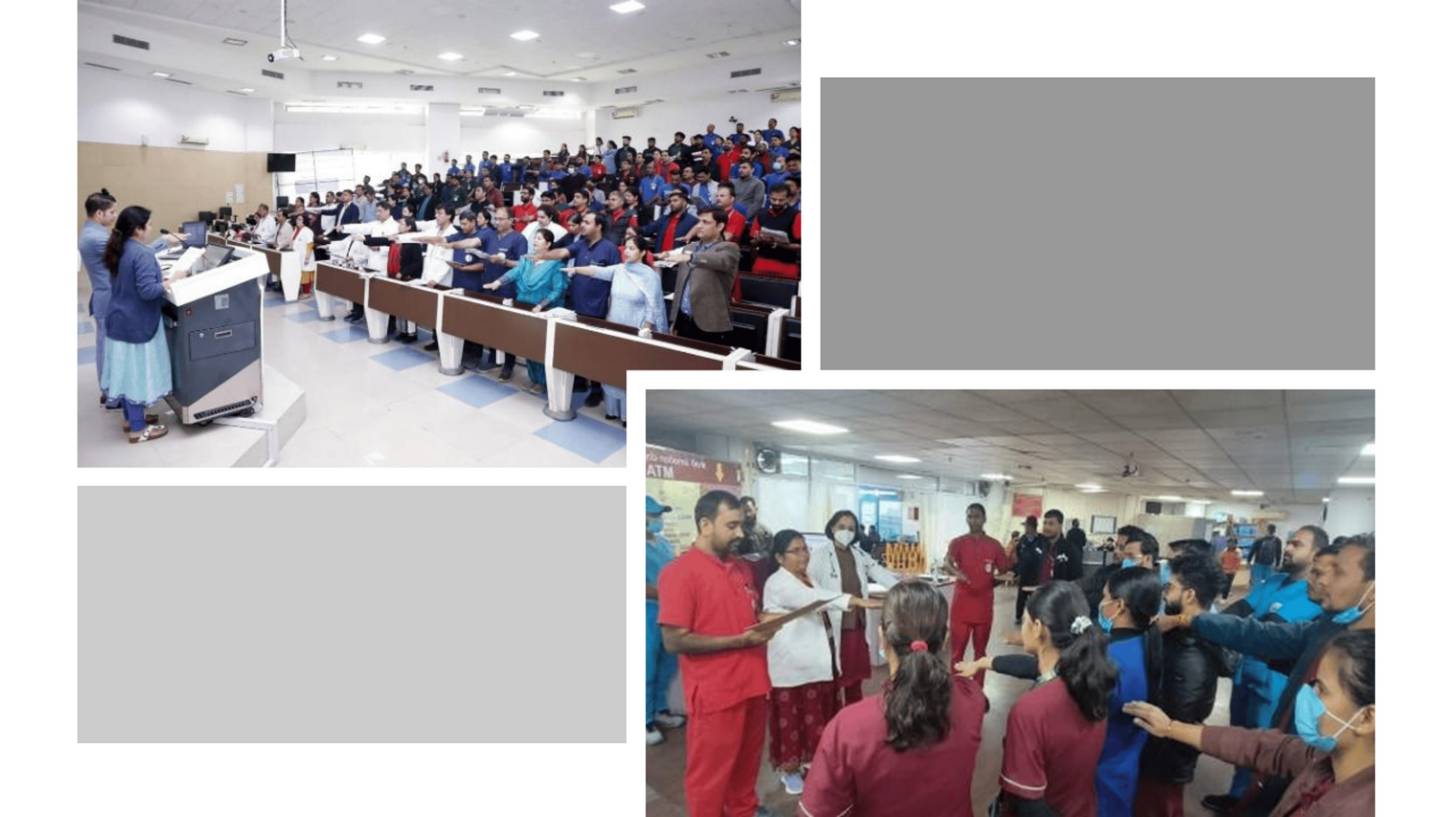
Oath ceremony in various sessions Cultivating institutional accountability: Multi-session Antimicrobial Stewardship (AMS) pledge ceremonies. This figure illustrates the synchronization of the AMS oath across diverse clinical and academic tiers. These ceremonies served as a critical behavioral intervention, formalizing the transition from individual clinical autonomy to collective institutional responsibility in the containment of antimicrobial resistance (AMR).

The WAAW 2024 activities successfully engaged a diverse group of stakeholders, with an estimated total participation exceeding 3000 individuals across all seven days on this IAP (Table 1). IAP was organized by >200 HCWs including all major admins of the hospital. The oath ceremony alone was administered to over 2000 HCWs across various sessions (Figure 1). 

Institutional Policy and Practice

**Figure 2:**
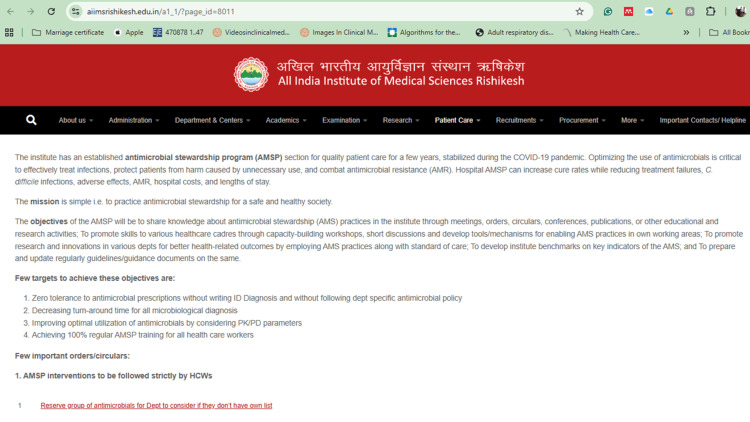
Administrative stewardship practice Operationalizing administrative stewardship: This serves as the foundational "enabling environment," ensuring that clinical guidelines are backed by institutional authority, resource allocation, and accountability mechanisms. This top-down commitment is essential for the long-term sustainability of the Institutional Action Plan (IAP).

The week marked the institutional commitment through the formal release of four critical IAS policy documents, which were made available on the institutional website for all HCWs to practice as a part of AMSP practice (Figure 2).

The comprehensive IAS checklist utilized during the ice-breaking sessions served as an effective real-time audit tool, leading to the identification of strengths and weaknesses in departmental adherence. The neonatal intensive care unit (NICU) was identified as the overall IAS champion ward based on the final assessment score among all 43 clinical areas across the hospital. Simultaneously >1000 people participated during the oath-taking sessions.

Educational Outcomes

**Figure 3:**
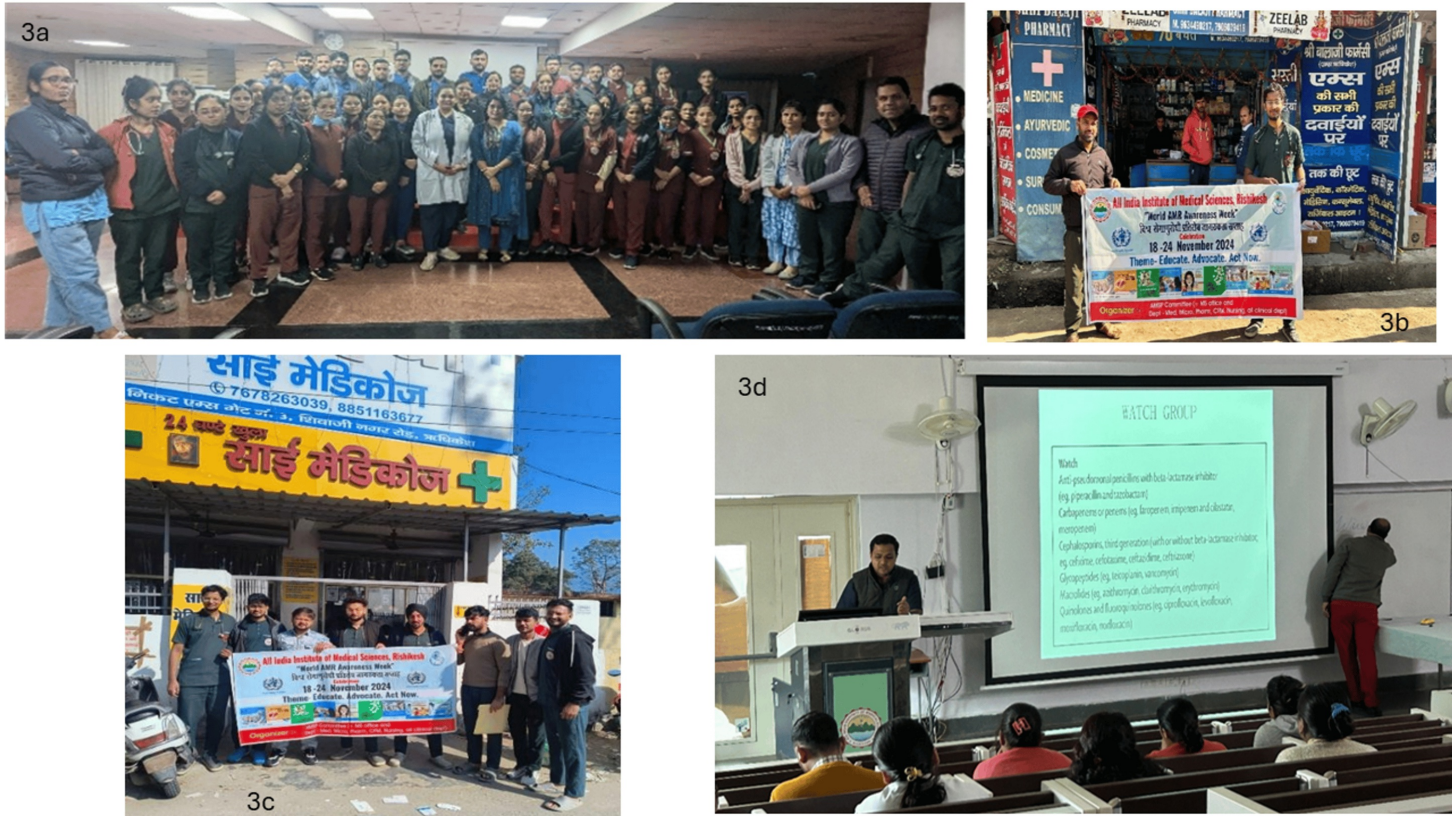
Multi-modal capacity building with interactive stewardship workshops 3a: Foundation workshop on Integrated Antimicrobial Stewardship Program (IASP); 3b, 3c, 3d: Pharmacy stewardship workshops.

Structured workshops provided targeted education to more than 300 HCWs (including consultants, nurses, residents, and pharmacists), focusing on basic stewardship principles (right sampling, clinical antibiograms, standard and transmission-based precautions, adult vaccination, AWaRe classification, PK-PD, IV-oral switch, OPAT) (Figures 3a-3d).

The special pharmacist workshop had 100 institutional and nearby pharmacy shop owners as participants.

**Figure 4:**
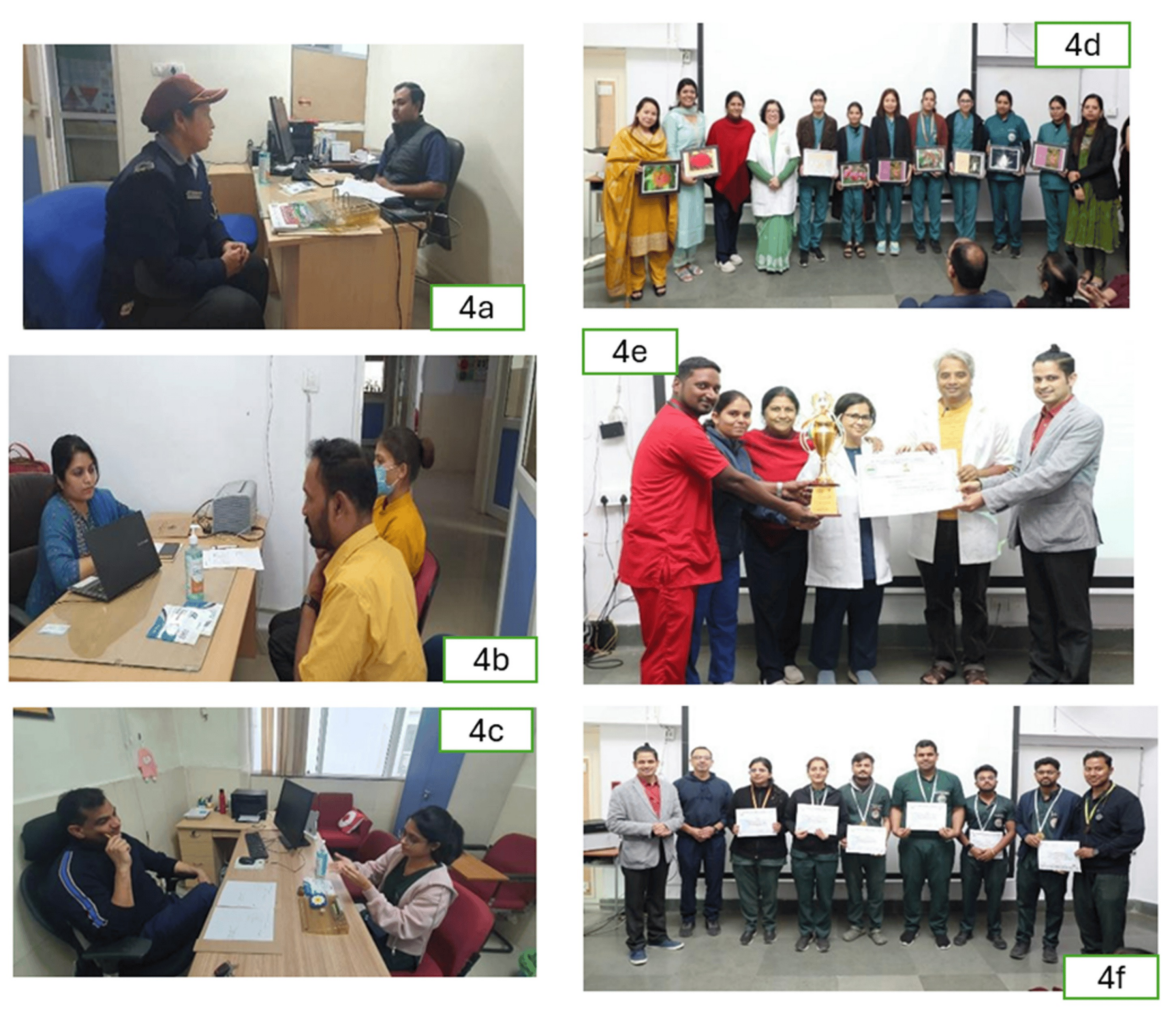
Behavioral incentivization with an Integrated Antimicrobial Stewardship (IAS) championship competition 4a: Interviewing guards; 4b: Interviewing hospital attendants/housekeeping (HA/HK) staff; 4c: Interviewing resident doctors; 4d: IAS championship award distribution to nursing staff; 4e: IAS championship award distribution to neonatal intensive care unit (NICU); 4f: IAS championship award distribution to resident doctors. Gamification of stewardship: The IAS championship was a motivational intervention. This figure captures the IAS competition, a strategic initiative designed to incentivize clinical excellence and adherence to the institutional IAS practice. By transforming compliance and clinical knowledge into a competitive framework, the Institutional Action Plan (IAP) model fosters a culture of "Positive Deviance," where top-performing departments and individuals are recognized as institutional benchmarks. This incentivization is a key driver for behavioral change and the long-term sustainability of the stewardship program.

The IAS championship competition effectively disseminated key IAS messages to a large number of HCWs, including consultants, resident doctors, interns, nurses, pharmacists, HAs, HK staff, security guards, and students (medical, nursing paramedical) who participated in the preliminary multiple choice question (MCQ) screening and then top six scorers in each category of HCWs were interviewed by a team of consultant experts to select the top two from each for prizes (Figures 4a-4f).

**Figure 5:**
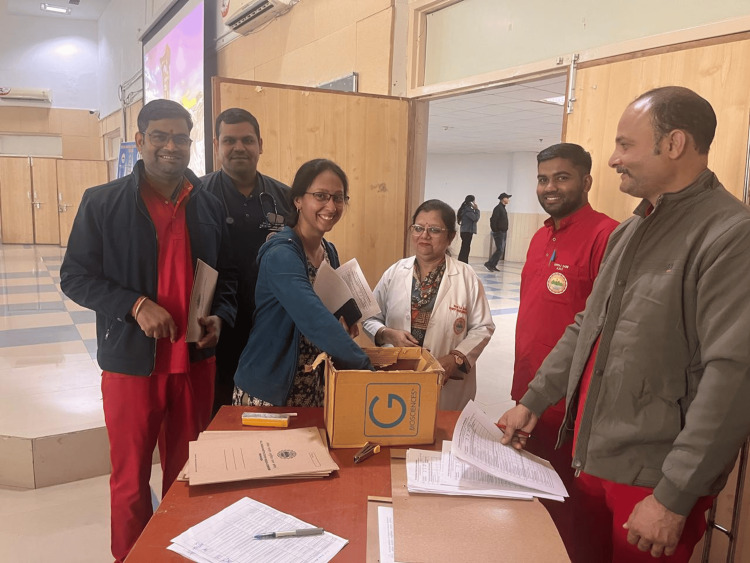
Bedside behavioral interventions via ward-based ice-breaking sessions Bridging the implementation gap with "Ice-breaking" bedside audit sessions. This figure illustrates the transition from classroom theory to ward-based practice through structured, non-punitive bedside audits. These sessions served as a "Situational Analysis" tool, allowing the antimicrobial stewardship (AMS) team to identify department-specific barriers to stewardship. By providing real-time, peer-to-peer feedback on current prescriptions and diagnostic samples, the sessions functioned as a behavioral "nudge," fostering immediate corrective actions and enhancing adherence to the institutional Integrated Antimicrobial Stewardship Program (IASP) checklist.

The hands-on component of the ice-breaking sessions resulted in immediate correction and reinforcement of IAS and documentation practices across 43 clinical areas. Each area's HCWs attended the session with an average participation of 20 per area, including the area nurse-in-charge. Based on the highest scores during this training-assessment sessions, six top areas were again trained and assessed for top three prizes (Figure 5).

Public Awareness Impact

**Figure 6:**
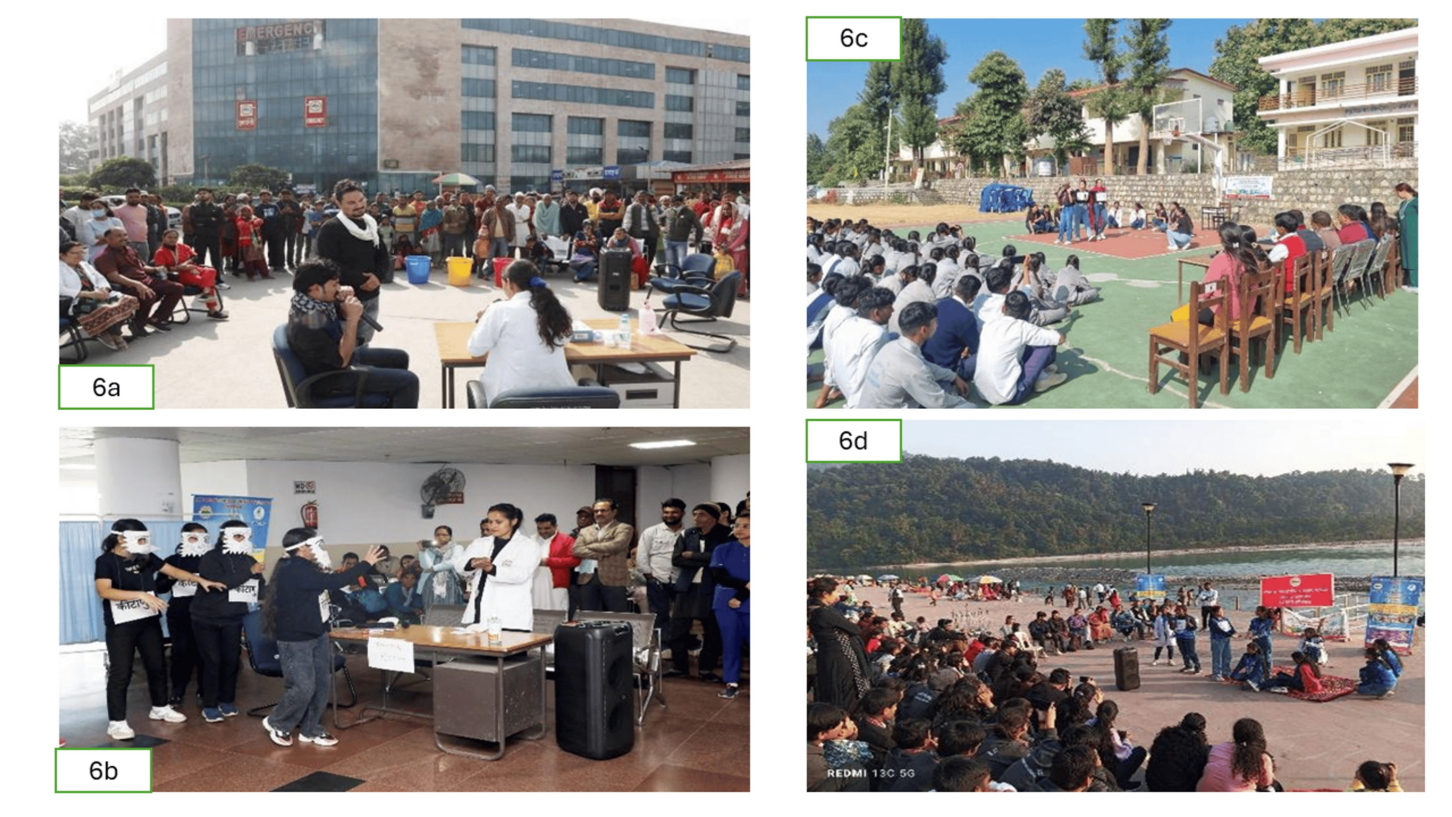
Roleplay Interventions for AMR 6a, 6b: Roleplays in busy out-patient department (OPD) areas; 6c: Roleplay in a government school; 6d: Roleplay in Triveni Ghat This figure depicts the use of narrative-based roleplays as a socio-behavioral intervention to address the drivers of antimicrobial resistance (AMR) in the community. By dramatizing the consequences of self-medication and non-compliance, the roleplays served as a "nudge" to alter public perception and health-seeking behaviors. This multimodal advocacy strategy aimed to bridge the gap between technical microbiological data and public health literacy.

Public awareness activities, especially the roleplays at the three OPD counters and Triveni Ghat, were highly successful in engaging the general public, reaching an estimated 1500 people (Figures 6a-6d).

**Figure 7:**
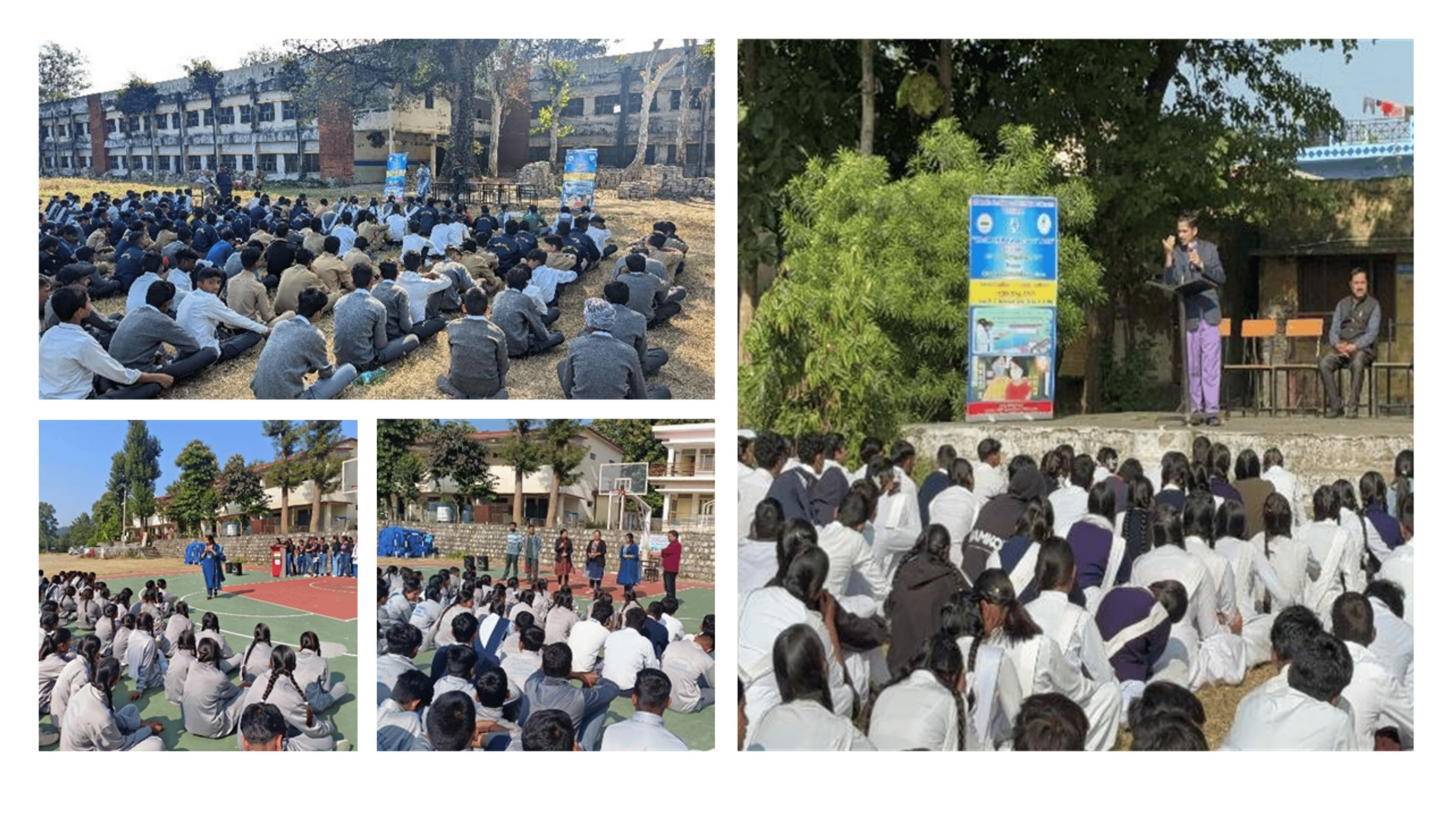
Public health literacy through awareness talks/sessions in local government schools Grassroots advocacy: Strengthening public health literacy via school-based interventions. This figure depicts the "Outreach" vertical of the World Antimicrobial Resistance Awareness Week (WAAW) 2024 Institutional Action Plan, targeting local government school students. By simplifying the science of microbes and the risks of antimicrobial misuse, these sessions aimed to create "AMR Awareness Ambassadors" within the community. This early-life intervention was a strategic component of the National Action Plan-Antimicrobial Resistance (NAP-AMR), designed to foster a culture of rational antibiotic use and hand hygiene that extends from the classroom to the household.

This format was observed to be highly effective in conveying complex public health messages to a non-medical audience. Outreach to three government schools resulted in the education of almost 800 students and staff, promoting the integration of IAS practices into daily life (Figure 7).

## Discussion

The findings herein demonstrate the successful and necessary transformation of the WAAW initiative from an isolated campaign into a robust, integrated, policy-driven AMS framework-the IAP model. This shift represents a significant, replicable advancement in the fight against AMR, directly addressing the critical implementation gap between national policy and localized clinical practice [12].

Strategic integration: policy, practice, and the one health mandate

The core strength of the AIIMS Rishikesh IAP model is its dual-axis approach, which strategically aligns all core objectives of the WHO's GAP-AMR [11] and India's NAP-AMR [13]. While the GAP-AMR mandates objectives spanning awareness, surveillance, IPC, and optimizing use, the institutional challenge lies in their unified execution. Our model achieved this by moving beyond voluntary awareness to mandated, structural change, thus enforcing long-term behavioral compliance and sustaining the 'boosting effect' of WAAW. Our program’s structure directly addresses the interconnected nature of the crisis, a key tenet of the 'One Health' concept [11].

Supply-Side Mandate (Healthcare/Prescriber Focus)

By integrating mandatory AMS policy, compulsory training, and audit feedback loops into the professional obligations of HCWs, the program ensured top-down adherence. This structural change aligned directly with the CDC’s AMR Solutions Initiative focus on strengthening domestic surveillance and infrastructure [3]. Such enforcement is a crucial distinction from voluntary or incentivized programs, which are often cited for variable compliance, especially in resource-limited settings [7]. Enforcing standardized, evidence-based prescribing acts as a potent measure against the rising tide of MDROs [8].

Demand-Side Engagement (Community/Patient Focus)

Parallel, sustained, and high-impact community outreach is vital for managing patient expectations and reducing the documented global issue of inappropriate over-the-counter antimicrobial use and self-medication, which significantly fuels resistance outside the hospital setting [6,10]. Sustained community engagement is the sine qua non for lasting behavioral change, ensuring that the progress made within the hospital walls is not undermined by external misuse, a concern specifically highlighted in the context of high antimicrobial consumption observed in India [12].

Sustainability, Replicability, and Global Impact

**Figure 8:**
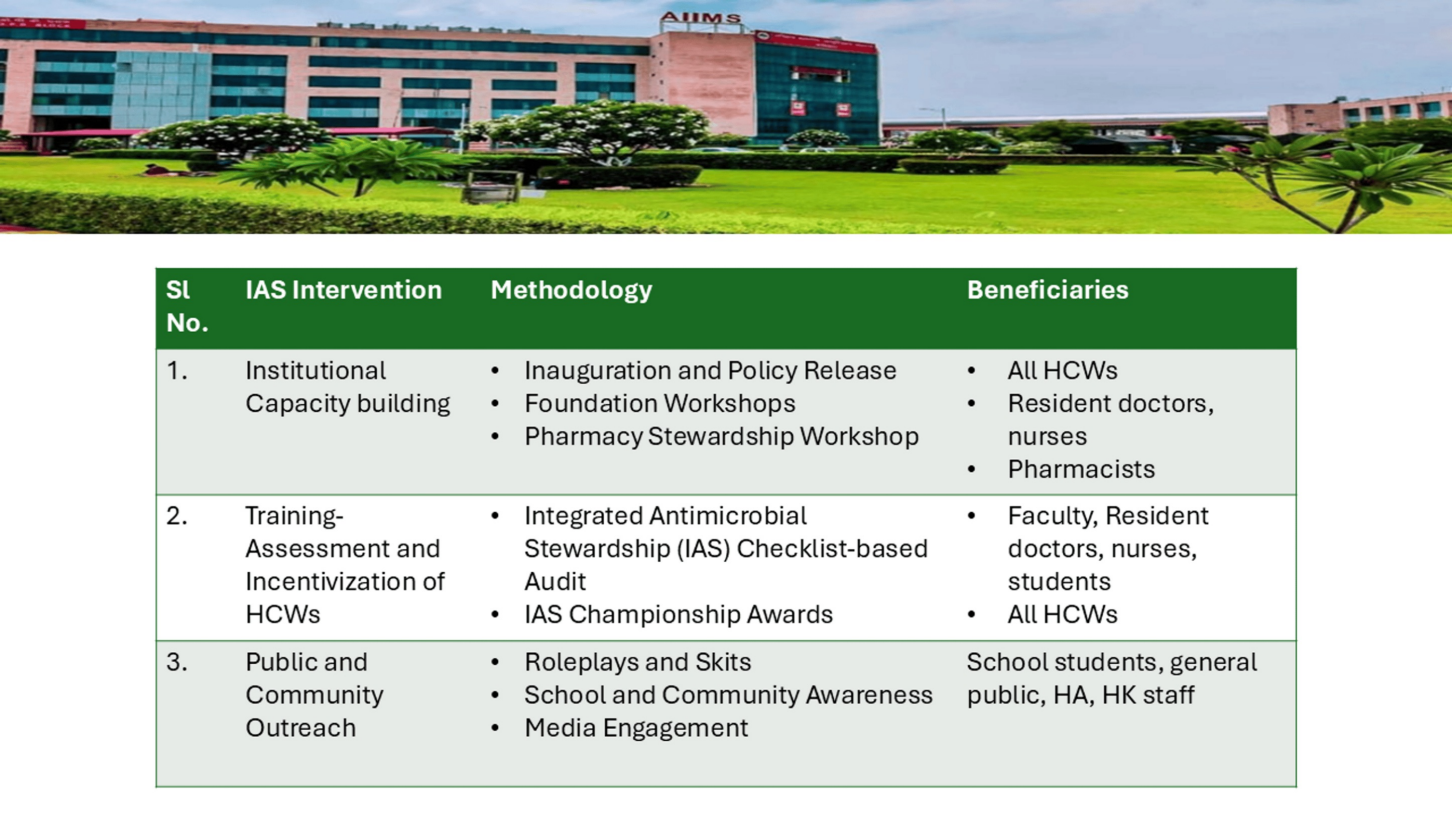
Implementation science via the IAP template of All India Institute of Medical Sciences (AIIMS) Rishikesh A replicable framework for institutional Antimicrobial Resistance (AMR) governance. This figure presents the comprehensive Institutional Action Plan (IAP) template designed to synchronize multi-sectoral efforts during World Antimicrobial Resistance Awareness Week (WAAW). As a "Sentinel Site" output, this template is structured for scalability, allowing other tertiary-care centers to adapt these interventions. HCW, Healthcare workers; HA, hospital attendant; HK, housekeeping.

The successful emphasis on high-level administrative support and genuine cross-sectoral collaboration is arguably the most important element validating the claim of long-term sustainability. The IACG report to the UN emphasized that effective AMR mitigation requires continuous vigilance and dedicated resources, positioning it as an existential threat alongside climate change [10]. The institutionalization of the WAAW framework within a major tertiary care center like AIIMS Rishikesh, with its complex patient demographics and elevated prevalence of MDROs, provided a robust IAP template (Figure 8).

Tertiary hospitals serve as essential proving grounds. Prior studies, even as far back as 2017/2018, have demonstrated that robust AMS interventions in Indian tertiary care settings, such as guideline implementation, audits, and data-driven feedback, are clinically effective, economically viable, and lead to measurable outcomes like reduced length of stay (LOS) and decreased antimicrobial expenditure [9].

By providing a clear, evidence-based IAP model, this initiative directly contributes to the global effort to meet the ambitious target set by the UNGA for a 10% reduction in human deaths related to bacterial AMR by 2030 [14]. Success here translates directly to a high potential for replication across other public and private hospitals, globally, offering a structured path forward for operationalizing high-level policy. This structured approach, grounded in policy-driven compliance and sustained community engagement, is necessary to combat the 1.27 million direct deaths attributed to AMR worldwide [8].

Limitations and future directions

While the program’s success in policy implementation and engagement is clear, the current data reflects process indicators (training completion, policy release, engagement metrics) rather than definitive outcome measures. This is a common pattern in the early stages of AMS program evaluation in the country. A critical next step is to translate this early success into verifiable clinical and microbiological impact. Future research must focus on tracking longitudinal data, specifically: (i) Antibiotic consumption density: measuring reduction in defined daily doses (DDDs) or days of therapy (DOT) per 1,000 patient-days, an established metric for AMS success; (ii) Microbiological shifts: documenting changes in resistance patterns for key indicator organisms; (iii) Clinical outcomes: Assessing the impact on healthcare-associated infection (HAI) rates and patient mortality.

## Conclusions

The WAAW 2024 program at AIIMS Rishikesh successfully transitioned from a collection of awareness events to a structured, integrated framework for policy implementation and >3000 participations of HCWs and public through this IAP model. By simultaneously empowering HCWs through mandated training and policy release, while actively engaging the community through high-impact outreach, this program established a robust and replicable model for IAS implementation at a tertiary care level. The high level of cross-sectoral engagement and administrative support confirmed the institution's capacity to sustain these efforts, critically contributing to the national effort to meet the global targets for AMR reduction. This AIIMS Rishikesh IAP model may be adapted in other tertiary care centers in the world, and AMR disasters can be prevented with time. It demonstrated that the global threat of AMR can be met with integrated, policy-backed action at the grassroots level of healthcare delivery. Scaling this replicable model across other facilities is a necessary step toward critically contributing to the national and global efforts to contain the spread of AMR.

## Appendices

**Table 3:** Ice break sessions: Assessment checklist cum training of HCWs on integrated antimicrobial stewardship (ISP/DSP/ASP) practices ID, Infectious Diseases; HCW/SNO, Health-Care Worker/ Senior Nursing Officer; PPE, Personal Protective Equipment; BMW, Bio Medical Waste; ICN, Infection Control Nurse; HAI, Hospital Acquired Infections; ISP/DSP/ASP, Infection Prevention Stewardship Practices/Diagnostic Stewardship Practices/Antimicrobial Stewardship Practices; IV, Intravenous; HICC, Hospital Infection Control Committee.

S.No.	ISP/DSP/ASP Questionnaires	Yes (1)	No (0)	Partial (0.5)	Not Applicable/Any comment
I	Assessment of knowledge, practice and audit on integrated antimicrobial stewardship (IAS) Practices of the local admins				
1.	Availability of ID practice document released in 2018 & 2022?				
2.	Availability of Institute Antibiotic policy 2022?				
3.	Availability of Antimicrobial utilization form in patients who are >5days of Antimimcrobials?				
4.	Availability of Uses of time dependent antimicrobial policy ?				
5.	Availability of Dept specific Reserve list of antimicrobials and its utility policy?				
II	Assessment of knowledge, practice and audit on infection prevention stewardship (ISP) Practices				
	Knowledge assessment by asking any HCW/SNO				
6.	When to use PPE ?				
7.	Any random HCW list standard precautions components ?				
8.	HCW enlist 5 moments of hand hygiene ?				
9.	Any available Protocol on soiled linen items ?				
10.	How to prepare various concentration of sodium hypochlorite solution ?				
11.	Aware about procedure for bed sore assessment and management in the unit.				
	Observation (No need to ask, just see each corner of area, sometimes ask SNO)				
12.	General or environment cleaning (Linen, beds, floor, walls, machines, door, BMW bins etc.)				
13.	Hand hygiene moments are followed in the right way				
14.	Availability of hand hygiene facility and soap solution in the ward				
15.	Facility for isolation room in the unit				
16.	Availability of signages boards in the unit				
17.	Availability of posters on needle stick injury (NSI), Hand hygiene etc.				
18.	Do the bundle care protocols are followed in unit (Refer Patient files or see for availability of checklist in the ward)				
19.	Hand rubs are available on every bed, nursing station, crash cart etc.				
20.	Do the catheter care is performed (Refer patient file)				
21.	Availability of infection control material like cleaning items, disinfection, PPE’s, etc. ( Verify from ward store)				
22.	Spill management kit is available in the area				
23.	Reporting (with in 3 months) of occurrence of HAI rate or surveillance data by HCWs or ICN (Refer from records/ Registers)				
24.	Dedicated color-coded BMW containers with lids and proper BMW segregation within the department				
25.	Emptying the BMW polythene cover on time when 1/3 is filled				
26.	Appropriate reporting and documentation mechanism for NSI in the ward : Check report/registers				
27.	Prompt health education for patients and patient family on infection control practices : Cross-verification with documentation				
28.	Area ICNs perform root cause analysis for HAI (Refer from records/ reports)				

S.No.	ISP/DSP/ASP Questionnaires	Yes (1)	No (0)	Partial (0.5)	Not Applicable/Any comment
III	Assessment of skills, knowledge, and auditing of Diagnostic Stewardship (DSP) procedures of HCWs				
	Knowledge Assessment				
29.	HCW understand the meaning of turnaround time (TAT) for an investigation?				
30.	HCW practices the administration of antibiotics following the withdrawal of the patient's sample, particularly culture (Refer File/ Records)?				
31.	HCW is aware of routine and emergency lab diagnostic testing and their implications in patient management?				
32.	Before drawing the sample, HCWs educate and explain the patient and caregivers the purpose and steps involved in a lab diagnostic test (Refer patient file/ Records)?				
33.	HCW document the investigation reports and updates diagnosis in the patient file (Assessment from patient file)?				
	Observations				
34.	Is the sample collection area clean? Are there any waiting samples there?				
35.	When the correct diagnosis is not being made, the report is not generated promptly, or the results are unclear, do the HCW consults clinical microbiology, biochemistry, pathology, radiology, or other specialists ?				
36.	Whether procedure room is organized and clean ?				

S.No.	ISP/DSP/ASP Questionnaires	Yes (1)	No (0)	Partial (0.5)	Not Applicable/Any comment
IV	Assessment of HCW's skills, knowledge, and auditing of antimicrobial stewardship (ASP) procedures				
37.	HCW comprehends and documents and practice one of the five Ds for a patient: right drug, dosage, duration, delivery, and decision on follow-up choice (Verification form records/ file)?				
38.	HCW understand and document the responses or evaluation of antimicrobial therapy (Verification from records/ file) ?				
39.	HCW does/reminds IV to oral switch timely of antimicrobials (Verification from records/ file)?				
40.	HCW does/reminds for STOP order after completion the course of antimicrobials (Verification from records/ file)?				
41.	HCW is aware of and utilizes the Antimicrobial Authorization Form (>5days on antibiotics) and the AWaRe checklist (Verification from records/file)?				
42.	During hospitalization and at the time of discharge, HCW teaches patients and caregivers about all aspects of antibiotics, particularly their side effects, precautions, etc. (Verification from records/ file)?				
43.	Do the HCW is aware the policy and how to dispose the expired antimicrobials ?				
44.	Do the HCWs is aware and provides regular feedback to the antimicrobial stewardship committee, HICC, and drug and therapeutic committee of the institute?				
